# Establishing minimal clinically important differences for the Quality of Life Instrument of Chronic Gastritis QLICD-CG(V2.0) based on distribution-based methods

**DOI:** 10.1186/s12876-023-02777-5

**Published:** 2023-05-12

**Authors:** Xiaoyu Wu, Ying Chen, Chonghua Wan, Lei Yu, Pingguang Lei, Xiaoyuan Sun

**Affiliations:** 1 School of Humanities and Management, Research Center for Quality of Life and Applied Psychology, Key Laboratory for Quality of Life and Psychological Assessment and Intervention, Guangdong Medical University, Dongguan, 523808 China; 2 School of Public Health, Kunming Medical University, Kunming, 650500 China; 3 Huadu District People’s Hospital Affiliated to Southern Medical University, Guangzhou, 510800 China; 4 People’s Hospital of Songgang, Baoan, Shenzhen, 518105 Guangdong China

**Keywords:** Chronic gastritis, Quality of life, Minimal clinically important difference, Distribution-based method

## Abstract

**Background:**

To establish the lowest score reflecting meaningful changes from the perspective of patients is very important for explaining the results of patient reports. The measurement scale of quality of life in patients with chronic gastritis has been used in clinical practice, but the minimal clinically important difference (MCID) has not been worked out. In this paper, we use a distribution-based method to calculate the MCID of the scale QLICD-CG (Quality of Life Instruments for Chronic Diseases- Chronic Gastritis) (V2.0).

**Methods:**

The QLICD-CG(V2.0) scale was used to evaluate the quality of life in patients with chronic gastritis. Since the methods for developing MCID were diverse and there was no uniform standard, we took MCID developed by anchor-based method as the gold standard, and compared the MCID of QLICD-CG(V2.0) scale developed by various distribution-based methods for selection. Standard deviation method (SD), effect size method (ES), standardized response mean method (SRM), standard error of measurement method (SEM) and reliable change index method (RCI) are given in the distribution-based methods.

**Results:**

A total of 163 patients, with an average age of (52.37 ± 12.96) years old, were calculated according to the various methods and formulas given by the distribution-based method, and the results were compared with the gold standard. It was suggested that the results of the SEM method at the moderate effect (1.96) should be taken as the preferred MCID of the distribution-based method. And thus the MCID of the physical domain, psychological domain, social domain, general module, specific module and total score of the QLICD-CG(V2.0) scale were 9.29, 13.59, 9.27, 8.29, 13.49 and 7.86, respectively.

**Conclusions:**

With anchor-based method as the gold standard, each method in distribution-based method has its own advantages and disadvantages. In this paper, 1.96SEM was found to have a good effect on the minimum clinically significant difference of the QLICD-CG(V2.0) scale, and it is recommended as the preferred method to establish MCID.

## Introduction

Chronic gastritis is a chronic gastric mucosal inflammation with a high incidence, and its incidence accounts for the first place in all kinds of gastric diseases. The clinical manifestations of patients are upper abdominal pain, nausea, and abdominal distension [1]. Due to the long course of disease, the patient's condition is easy to delay, and the radical treatment is difficult, and it is closely related to gastric cancer [2], the long-term efficacy of patients with chronic gastritis is not ideal. Chronic gastritis adversely affects the quality of life (QOL) of patients and threatens their physical and mental health. To determine the therapeutic effect, it is crucial to accurately assess subjective feelings.

Quality of Life (QOL) is defined by the WHO Quality of Life Research Group as the experience of individuals in different cultures and value systems of living conditions in relation to their goals, aspirations, standards and concerns [3]. This is a concept with extensive connotation. In the medical field [4], from the perspective of health and medical care, the research on quality of life is limited to a certain range, which mainly refers to the evaluation of the physiological, psychological and social functions of individuals, namely, health-related quality of life (HRQOL). Quality of life measurement tools are regarded as important contributions to clinical practice. To detect and quantify health status, a variety of generic and specific tools have been developed [5, 6]. The generic scale is suitable for the assessment of different diseases and population interventions, but the response to assess QOL of patients with specific diseases or to capture minor changes in patients is poor. For evaluating the quality of life of patients with chronic gastritis, the disease-specific scale is more suitable than the generic scale at this time. In order to evaluate QOL of patients with digestive diseases, several disease-specific scales have been developed. For example, gastrointestinal symptom rating scale (GSRS) [7], gastrointestinal quality of life index (GIQLI) [8] and nepean dyspepsia index (NDI) [9], but these scales are not scales for specific symptoms of chronic gastritis. Moreover, the development of these scales is not based on the current popular modular development method, which is composed of the general module and specific modules. It is well known that the modular approach is helpful to capture common diseases and unique disease characteristics for the general module can be used to compare QOL across different diseases while the specific module can be used to depicture symptoms and treatments in detail.

Consider combining generic and disease specificity in the questionnaire and responding directly to the need for chronic gastritis, we have developed a chronic gastritis scale under the system of Quality of Life Instruments for Chronic Diseases, QLICD-CG(V2.0) [10].

When we applied the scale, we discovered, however, that determining the therapeutic effect required a parameter. The minimal clinically important difference (MCID) is used to determine the extent to which health assessment tool changes are clinically relevant. The MCID is the minimum amount of score change. From the perspective of patients, these changes can be considered as meaningful [11]. In 1989, Jaeschke et al. formally defined MCID as the minimum change value that patients thought was beneficial without considering side effects and cost burden, which could promote the change of patient management scheme [12]. Through the understanding of MCID, meaningful explanation of research results is very important for the research and practice of treatment results.

MCID can be calculated in a number of ways, but there is no consensus on the best method to use. At present, the methods of calculating MCID are generally divided into anchor-based method and distribution-based method. Anchor-based method calculates the degree of excess of accidental or random fluctuation by comparing the change of patient score with the anchor of the same patient. In contrast, the distribution-based method uses statistical indicators based on the change of the whole population score to calculate the minimum change of real change or excess of random fluctuation, which focuses on the score difference between patients [13]. As far as the distribution-based method is concerned, a large number of articles [14, 15] have proposed specific calculation methods, but the methods are also diverse, and there is no standard to determine which method is more specific to the true changes of patients [16]. In view of the fact that the QLICD-CG(V2.0) scale has no MCID available for reference, this paper intends to explore various calculation methods in the distribution-based method through the analysis and comparison of actual data, and develop appropriate MCID, this also provides a basis for the future use of distribution-based methods.

## Methods

### Settings and participants

Patients with chronic gastritis diagnosed in the inpatient departments of gastroenterology, such as Affiliated Hospital of Guangdong Medical University and Shilongboai Hospital of Dongguan City, from November 2012 to September 2014 were selected as the participants.

Inclusion criteria: (1) patients diagnosed as chronic gastritis by gastroscopy or gastric mucosal biopsy; (2) participants who have certain reading, writing, and understanding skills and voluntarily participate in the survey.

Exclusion criteria: (1) patients with illiteracy and lack of literacy; (2) patients with other serious diseases; (3) patients unwilling to cooperate. The institutional review committee approved the study, and the respondents were voluntary and provided written “ informed consent” for participation.

### Investigation tools

The scales of this study include QLICD-CG(V2.0) scale and The Medical Outcomes Study 36-Item Short-form (SF-36 scale). QLICD-CG(V2.0) scale consists of two parts: the general module (28 items) and the specific module (11 items). The general module includes three domains of physical function, psychological function and social function, and the specific module includes three facets of epigastric pain, satiety and psychological impact for chronic gastritis. QLICD-CG(V2.0) has been used in clinical practice, and the verification of data [10, 17] shows that the scale has good psychometric properties, and it can be used as a useful tool for assessing QOL in patients with chronic gastritis (The QLICD-CG(V2.0) scale was validated and confirmed good psychometric properties, with the Cronbach ' s alpha coefficient of each domain and the total score of the scale being 0.80–0.93, split-half reliability > 0.66, test–retest reliability > 0.80. The differences in various domains and the total score of the scale before and after the intervention were statistically significant with moderate or higher effect size SRM (standardized response mean). The entire scale has a total of 39 items, each of which rated as a 5-point Likert rating system.

SF-36 scale [18] is a general tool to measure health-related quality of life. It includes 36 items, which are used to measure eight domains. These domains are scored from 0 to 100 following a standard algorithm with higher scores representing better QOL.

### Survey methods

In the whole process of the investigation, the investigators appeared as a clinical doctor. They first gave a brief description of the purpose of the investigation to patients with chronic gastritis who met the inclusion criteria. After obtaining the consent, the patients were asked to fill out the QLICD-CG(V2.0) and SF-36 according to their actual situation. After completing the scale, the scale was immediately retracted, and whether there were any missing items was checked. If there were, the patients were asked to fill in to make it complete. Three surveys were conducted on the same hospitalized patients. The first measurement was carried out on the first day of admission, which was the measurement before treatments. The second measurement was carried out on the second day after admission for evaluation of test‐retest reliability, and the third measurement was carried out on the day before discharge after treatments. The data from first and third measurements are used to make out MCID in this research.

### Scoring methods

After the data was collected, Epdata3.1 software was used for data collation and SPSS22.0 software was used for data analysis. For the score of items, each item is a five-level hierarchy, which is calculated as l, 2, 3, 4 and 5 points in turn. Positive statement items are rated directly from 1 to 5, while negative statement items are scored in reverse. The domain and overall scores are obtained by adding related item scores, all of which are linearly converted to standardized scores on a scale of 0‐100 [19].

### MCID calculation methods

In the selection of MCID calculation method, the distribution-based method which takes into account the sampling error and does not need to select the external anchor has attracted the attention of researchers. Because there is no fixed calculation method for MCID, this paper takes the anchor-based method as the gold standard, the distribution-based method is used to calculate the MCID, and the accuracy of the distribution-based method is compared with the gold standard.

### Gold standard by anchor-based method

Some studies [20] pointed out that MCID can be determined only when the linear correlation coefficient between calibration and quality of life or clinical efficacy score is not less than 0.30. According to the first item Q1 in the SF-36 scale “overall, your health: 1. Excellent, 2. very good, 3. good, 4. fair, 5. poor”was subjective calibration, and the differences in Q1 score (score increased or decreased) before and after intervention were screened out. X0 represents baseline score of the respondent (on the day of admission), X1 represents post-intervention score of the respondent (on the day before discharge), and then the difference d of two standardized measurement scores of the same patient is determined. Then, the mean of the difference ($\left|\overline{d }\right|=\frac{d1+d2..d\mathrm{n}}{n}$) is taken as MCID. If the difference obeys skewed distribution, the median (n is odd, MCID$={d}_{\frac{n+1}{2}}$, n is even, MCID$=\frac{1}{2}\left({d}_\frac{n}{2} ,{d}_{\frac{n}{2}+1}\right)$) is taken as MCID. 

### Distribution-based methods

The distribution-based methods are based on the statistical parameters of large sample data to calculate the MCID, which have a clear formula and are easy to calculate. In the distribution-based methods, seven methods such as standard deviation method (SD), effect size method (ES), standardized response mean method (SRM), standard error of measurement method (SEM), reliable change index method (RCI), and growth curve analysis were used to determine the MCID. The specific formula is shown in Table 1.

1. Standard deviation method (SD) [21]: SD describes the variation or dispersion of a set of data values. Generally, 0.5SD of baseline of data is taken as MCID.
2. Effect size method (ES) [22]: ES represents the ratio of the difference in mean scores before and after intervention to the standard deviation of scores before intervention.
3. Standardized response mean method (SRM) [23]: SRM is the ratio of the difference between the mean score of the scale before and after the intervention and the standard deviation of the score difference before and after the intervention, which is an index to measure the effect of score change.
4. Standard error of measurement method (SEM) [24]: SEM is an index to measure the change of fraction observed due to the measurement error compared with the real fraction. SEM shows the minimum variation higher than the measurement error, which is considered to be the characteristic of measurement.
5. Reliability change index method (RCI) [25]: The calculation method of RCI is to divide the patient ' s individual change score by the square root of SEM, which is used to evaluate the change range necessary for the statistical reliability of the given self-report measurement.

**Table 1:** Calculation formulas for distribution-based methods

Method of calculation	Formula	Variable declaration
Standard deviation method (SD)	$S{D}_{baseline}=\sqrt{{\sum \left({x}_{0}-{\overline{x} }_{0}\right)}^{2}/\left(n-1\right)}$ $MCID=0.5*\text S{D}_{basline}$	${x}_{0}$ is the baseline score for patients before intervention, $\overline{{x }_{0}}$ is the mean score of patients before intervention, and n is the sample size, $S{D}_{baseline}$ is the standard deviation of score before intervention. MCID is 0.5SD of baseline data.
Effect size method (ES)	$ES=\frac{\overline{{x }_{1}}-\overline{{x }_{0}}}{\sqrt{\sum {({x}_{0}-\overline{{x }_{0}})}^{2}/(n-1)}}$ $MCID=ES*S{D}_{baseline}$	$\overline{{x }_{1}}$ is the mean score after intervention. MCID is the calculation result when the baseline data is 0.2, 0.5, and 0.8 respectively.
Standardized response mean method (SRM)	$SRM=\frac{\overline{{x }_{1}}-\overline{{x }_{0}}}{\sqrt{\sum {({d}_{i}-\overline{d })}^{2}/(n-1)}}$ $MCID=\text SRM*S{D}_{d}$	${d}_{i}$ is the change of scores before and after intervention, $\overline{d }$ is the mean of the changed score. MCID was calculated when the data were 0.2, 0.5 and 0.8 after intervention.
Standard error of measurement method (SEM)	$SEM=\text{}\sqrt{1-r*S{D}_{basline}}$ $MCID=SEM*X$	r is the reliability coefficient, generally using test–retest reliability coefficient. If the test–retest reliability coefficient is unknown, the Cronbach coefficient can be replaced.
Reliability change index method (RCI)	$RCI=\sqrt{2*SEM}$ $MCID=X*S{D}_{baseline}*\sqrt{2(1-r)}$	

In the ES and SRM methods, it is considered that 0.2 is the low difference, 0.5 is the medium difference, and 0.8 is the high difference [26]. In the SEM and RCI methods, it is considered that 1 is the low difference, 1.96 is the medium difference, and 2.77 is the high difference [27].

### Quality control

Quality control is an important guarantee for the quality of research, and this paper adopts the following quality control measures: (1) specialized training of investigators is carried out before the investigation, and the purpose, method and content of the investigation, the description of the indicators and the way of questioning are mastered; (2) in the process of on-site investigation, check and review the questionnaire in a timely manner and correct the omissions and errors immediately; (3) the data were input by two personnel, and then the two input data were checked one by one, and the input error was found and corrected in time. Cases with incomplete records of main analysis indicators were excluded.

## Results

### Socio-demographic characteristics of the sample

In this study, 163 cases received the first survey with the age being 52.37 ± 12.96, 157 cases received both the first and third surveys. The basic information is shown in Table 2.

**Table 2:** Socio-demographic characteristics of the sample (*n* = 163)

Items	Case load(n)	Constituent ratio(%)	Items	Case load(n)	Constituent ratio(%)
**gender**			**nation**		
male	74	45.4	han nationality	162	99.4
female	89	54.6	other	1	0.6
**age**			**Family economic status**		
20–40	29	17.8	poor	49	30.1
40–60	88	54.0	medium	101	62.0
≥ 60	46	28.2	good	13	8.0
**degree of education**			**medical form**		
primary school	50	30.7	self-supporting	9	5.5
junior high school	55	33.7	Social medical insurance (urban and worker health insurance)	107	65.6
High school or technical secondary school	38	23.3	commercial health insurance	6	3.7
junior college	12	7.4	cooperative medical care	36	22.1
bachelor degree or above	8	4.9	missing data	5	3.1
**occupation**			**clinical diagnosis**		
worker	24	14.7	superficial type	52	31.9
farmer	59	36.2	the superficial type with erosion type	33	20.2
teacher	9	5.5	flat erosion	67	41.1
cadre	11	6.7	bile reflux type	3	1.8
private ownership	17	10.4	complex style	1	0.6
other	43	26.4	missing data	7	4.3
**marriage status**			**clinical stages**		
unmarried	7	4.3	asymptomatic period	4	2.5
married	148	90.8	symptom phase	155	95.1
divorced	3	1.8	missing data	4	2.5
widowed	5	3.1			

It can be seen from Table 2 that the proportion of male and female patients is similar; the majority is of Han nationality, mostly married. In terms of self-assessment of family economic situation, most of them choose the economic situation “middle”. Medical forms are mainly medical insurance for urban workers and cooperative medical care. The occupation is mainly farmers, followed by others. On educational level, patients are mainly in junior high school, followed by primary school.

### MCID calculated by anchor-based method

The correlation between Q1 item and each domain and total score of the scale was calculated. It was found that r > 0.30 (physical domain = 0.33, psychological domain = 0.20, social domain = 0.37, general module = 0.34, specific module = 0.36, total score = 0.38) in almost all domains, and the correlation between Q1 item and the scale was strong. Then, the score difference of each domain and the total score of the scale was calculated and it was found that, based on the Q1 item, the score change of patients had multiple levels: 1, 2 and 3 levels increased, and 1 level decreased. Among them, 79 patients increased by 1 level before and after intervention, 16 patients increased by 2 levels before and after intervention, 2 patients increased by 3 levels before and after intervention, 4 patients decreased by 1 level before and after intervention, 47 patients showed no change in Q1 item before and after intervention, and 15 patients showed missing value. *P* < 0.05, does not conform to normality, the median difference is the MCID of anchor-based method. The MCID values of each domain were 11.11, 9.09, 9.38, 9.82, 13.64 and 10.90 by anchor-based method. See Table 3 for details.

**Table 3:** MCID of the QLICD-CG determined by anchor-based method (*n* = 157)

Domain	Physical domain	Psychological domain	Social domain	General module	Specific module	Total score
Number of items	9	11	8	28	11	39
Number of cases	101	101	101	101	101	101
*t*^a^	-7.93	-7.21	-5.08	-8.05	-8.50	-9.00
*P*	0.00	0.00	0.00	0.00	0.00	0.00
MCID	11.11	9.09	9.38	9.82	13.64	10.90

^a^t test for normality of score difference before and after treatments

### MCID calculated by distribution-based methods

It was found that the retest reliability of physical domain, social domain, general module and total score of the scale were all 0.91, the retest reliability of psychological domain was 0.86, and the retest reliability of specific module was 0.83. According to the calculation results of various formulas given in Table 1, when SD = 0.5, the MCID of each domain were 7.69, 9.16, 7.76, 6.86, 8.32 and 6.61, respectively. ES = 0.2/0.5/0.8, the MCID of each domain were 3.08/7.69/12.30, 3.67/9.16/14.66, 3.10/7.76/12.41, 2.74/6.86/10.98, 3.33/8.32/13.32 and 2.64/6.61/10.57, respectively. SRM = 0.2/0.5/0.8, the MCID of each domain were 1.71/4.27/6.83, 2.14/5.35/8.56, 1.97/4.93/7.89, 1.51/3.77/6.03, 2.24/5.60/8.96 and 1.49/3.73/5.97, respectively. When SEM = 1/1.96/2.77, the MCID of each domain were 4.74/9.29/13.13, 6.93/13.59/19.20, 4.73/9.27/13.10, 4.23/9.27/13.10, 6.88/13.49/19.07 and 4.01/7.86/11.10, respectively. RCI = 1/1.96/2.77, the MCID of each domain were 6.70/13.14/18.57, 9.80/19.21/27.15, 6.69/13.11/18.53, 5.98/11.72/16.57, 9.74/19.08/26.97, and 5.67/11.11/15.70, respectively. See Table 4, 5 and 6 for details.

**Table 4:** MCID of the QLICD-CG determined by SD method and ES method

Domain	$S{D}_{baseline}$	0.5SD	0.2ES	0.5ES	0.8ES
Physical domain	15.38	7.69	3.08	7.69	12.30
Psychological domain	18.33	9.16	3.67	9.16	14.66
Social domain	15.51	7.76	3.10	7.76	12.41
General module	13.72	6.86	2.74	6.86	10.98
Specific module	16.65	8.32	3.33	8.32	13.32
Total score	13.22	6.61	2.64	6.61	10.57

**Table 5:** MCID of the QLICD-CG determined by SRM method

Domain	$S{D}_{d}$	0.2SRM	0.5SRM	0.8SRM
Physical domain	8.54	1.71	4.27	6.83
Psychological domain	10.70	2.14	5.35	8.56
Social domain	9.87	1.97	4.93	7.89
General module	7.53	1.51	3.77	6.03
Specific module	11.21	2.24	5.60	8.96
Total score	7.47	1.49	3.73	5.97

**Table 6:** MCID of the QLICD-CG determined by SEM method and RCI method

Domain	*r*	1SEM	1.96SEM	2.77SEM	1RCI	1.96RCI	2.77RCI
Physical domain	0.91	4.74	9.29	13.13	6.70	13.14	18.57
Psychological domain	0.86	6.93	13.59	19.20	9.80	19.21	27.15
Social domain	0.91	4.73	9.27	13.10	6.69	13.11	18.53
General module	0.91	4.23	8.29	11.72	5.98	11.72	16.57
Specific module	0.83	6.88	13.49	19.07	9.74	19.08	26.97
Total score	0.91	4.01	7.86	11.10	5.67	11.11	15.70

### Comparisoins of recommened MCID by anchor-based and distribution-based methods

Based on the anchor-based method, we calculated the MCID of the scale using various formulas in the distribution-based method, and found that the MCID of SD method was small when SD was 0.5. In ES method, 0.5 is the most appropriate result, while 0.2 or 0.8 will make the result too small or too large. In SRM method, the result of 0.8 is far less than that of anchor-based method. In SEM method, the MCID of 1.96 is closest to that of anchor-based method. In RCI method, when 1 is taken, the result is too small, and when 1.96 or 2.77 is taken, the result is too large. Considering the stability of the results, we recommend the MCID of each domain calculated by 1.96SEM method in the distribution-based method. See Table 7 for details.

**Table 7:** MCID of each domain of the scale calculated by anchor-based method and distribution-based method

Domain	anchor-based method	1.96SEM
Physical domain	11.11	9.29
Psychological domain	9.09	13.59
Social domain	9.38	9.27
General module	9.82	8.29
Specific module	13.64	13.49
Total score	10.90	7.86

## Discussions

There is no uniform standard to define which method of MCID is more in line with the actual situation of patients. The common methods for calculating the minimal clinically important difference include anchor-based method, distribution-based method, expert opinion method and literature analysis method. The expert opinion method is to determine the MCID according to the suggestions of clinicians and experts, and the literature analysis method is to determine the MCID according to the comprehensive analysis of existing literature. Each method has its own advantages and disadvantages. Anchor-based method pays attention to the feelings of patients, not only studies the design type, but it is difficult to select the appropriate anchor, and it is easy to produce memory offset [28]. The distribution-based method incorporates the error into the calculation and has a clear formula, but it depends on the statistical characteristics of the distribution and cannot reflect the patient’s point of view [29]. There is one-sidedness in expert opinion method and literature analysis method, which is rarely used in clinical practice.

Generally, anchor-based method is the preferred method given suitable anchor. Only when the anchor-based method cannot be implemented if there is no good anchor or the sample size is small, the distribution-based method is considered separately. In a study [27] on the formulation of MCID for systemic lupus erythematosus, it was pointed out that the distribution-based method was limited by their ability to define only the minimum value. When it was lower than the minimum value, the change in the score of the given measurement results might be due to the measurement error, which did not provide information about clinical importance. These methods largely separate the clinical importance of a given change in the defined outcome score from its statistical significance.

Due to the lack of gold standard for calculating MCID, this paper combines the anchor-based method with the distribution-based method, taking the anchor-based method as the gold standard. The main indicators in the distribution-based method are standard deviation (SD), effect size (ES), standardized response mean (SRM), standard error of measurement (SEM) and reliable change index (RCI). By comparing the MCID calculated by each index in the two methods of ES and SRM method, it is considered that 0.2 is low difference, 0.5 is moderate difference, 0.8 is high difference. No matter what kind of difference is taken to calculate MCID, the results of ES method are greater than those of SRM method. The calculation formulas of the two methods are similar. The only difference is that the denominator in the calculation formula of ES method is standard deviation of the score before intervention, and the denominator of SRM method is standard deviation of the score difference before and after intervention. Compared with SRM method, ES method is more vulnerable to the influence of baseline data standard deviation, which makes the overall value larger. Higher MCID may mistakenly classify patients with effective treatment as ineffective.

In regards to SEM and RCI methods, it is considered that 1 is taken as the low-level difference, 1.96 is the medium-level difference, and 2.77 is the high-level difference. No matter which difference is taken to calculate MCID, the results obtained by RCI method are greater than those obtained by SEM method. The use of SEM method to calculate MCID is more likely to overestimate therapeutic effects than RCI method. Mouelhi Y [30] found that the most common indicators in the distribution method included 0.2 SD, 0.3 SD, 0.5 SD and SEM.

According to the results of various methods, the MCID obtained by the 0.5ES method is smaller than that obtained by the gold standard (anchor-based method). In ES and SRM methods, the MCID of low difference (0.2) and moderate difference was smaller, and even the result of 0.8SRM method was still small. The low difference (1) and high difference (2.77) results obtained by SEM will make the clinical treatment effect deviate from the reality. In the RCI method, the MCID of low difference (1) was smaller, while the MCID of moderate difference (1.96) and low difference (2.77) would make clinical treatment considered ineffective. After referring to the literature [31, 32], it is suggested that the MCID calculated by the SEM method is preferred when the moderate difference is taken, which conforms to the characteristics that SEM is considered to be stable in different populations and different studies. There are two reasons: one is that it takes into account the statistical characteristics of the obtained scores, namely its importance, sample change or measurement accuracy, and is not seriously affected by sample size or variation [33]; second, it uses the reliability score of the questionnaire to determine its measurement error, and the calculation results show that the test‐retest reliability in various domains of the scale is high and has high reliability, which minimizes the fluctuation caused by measurement error by [34]. Consequently, the suggested MCID of physical domain, psychological domain, social domain, general module, specific module and total score of QLICD-CG(V2.0) scale were 9.29, 13.59, 9.27, 8.29, 13.49 and 7.86, respectively. This method is similar to a method for developing MCID for Crohn’s disease [35]. In another paper [36], the author used anchor-based method and distribution-based method to explore the MCID of breast cancer patients with one grade increase and at least one grade increase as two criteria. The distribution-based method used ES, SEM and RCI, and the results showed that the MCID values calculated by the two criteria of anchor-based method were similar. The results were 0.8ES, 1.96 SEM and 1.96 RCI, respectively.

In this study, SD method and ES method in the distribution-based methods are also compared using the anchor-based method as the standards. It is found that MCID calculated by 0.5ES method is similar to that calculated by anchor-based method, and thus MCID calculated by 0.5ES is suggested for ES method.

This study has certain limitations. First, different statistical indicators may produce different MCID. Secondly, the distribution-based method is limited by its ability. They can only define a minimum value below which the change in the score of a given measurement result may be due to measurement error, which does not provide information about clinical importance [21]. Moreover, the population characteristics and intervention measures of the subjects are different, and the MCID are different. Therefore, the current results cannot be generalized to other clinical environments. A small sample size may also affect the accuracy of MCID. However, although this study meets the basic requirements of MCID estimation, a larger sample size study is necessary to verify the current findings in the future.

In summary, we give different distribution methods and relevant MCID when we use the anchor-based method as the gold standard, and users can refer to the research purpose and sample characteristics to select one. Considering the stability, we recommend using 1.96 SEM method (taking the moderate differences) as the MCID of QLICD-CG(V2.0) scale, that is, the MCID of physical domain, psychological domain, social domain, general module, specific module and total score of QLICD-CG(V2.0) scale were 9.29, 13.59, 9.27, 8.29, 13.49 and 7.86, respectively.

## Conclusion

At present, there are a variety of methods for the formulation of MCID for the quality of life of patients with chronic gastritis and there is no universally recognized and unified standard. Therefore, we used anchor-based method as the gold standard and gave different distribution-based methods and MCID. It was found that 1.96SEM has a good effect on the minimal clinically important difference of the QLICD-CG(V2.0) scale, and it is recommended as the preferred method for the establishment of MCID.

## Data Availability

The data (two formats: SPSS and Excel) can be available by request from Prof. Chonghua Wan (Email: wanchh.hotmail.com).

## Abbreviations

QLICD-CG(V2.0): The quality of life instrument of chronic gastritis
MCID: The minimal clinically important difference
SD: Standard deviation method
ES: Effect size method
SRM: Standardized response mean method
SEM: Standard error of measurement method
RCI: Reliable change index method
SF-36: The medical outcomes study 36-item short-form
